# Correction: In vivo targeting of de novo DNA methylation by histone modifications in yeast and mouse

**DOI:** 10.7554/eLife.30948

**Published:** 2017-08-31

**Authors:** Marco Morselli, William A Pastor, Barbara Montanini, Kevin Nee, Roberto Ferrari, Kai Fu, Giancarlo Bonora, Liudmilla Rubbi, Amander T Clark, Simone Ottonello, Steven E Jacobsen, Matteo Pellegrini

Morselli M, Pastor WA, Montanini B, Nee K, Ferrari R, Fu K, Bonora G, Rubbi L, Clark AT, Ottonello S, Jacobsen SE, Pellegrini M. 2015. In vivo targeting of de novo DNA methylation by histone modifications in yeast and mouse. *eLife* **4**:e06205. doi: 10.7554/eLife.06205.

Published 7, April 2015

Recently, a colleague noticed that the data cannot be accessed using the GEO accession number reported in the published article.

We discovered that the GEO accession number was incorrectly reported as GSE6691. The GEO Accession number has been corrected to GSE66911.

This did not affect any results reported in the paper. The authors apologize for the mistake.

The article has been corrected accordingly.

