# Supplementary material for: In vivo targeting of de novo DNA methylation by histone modifications in yeast and mouse
Source: eLife. 2015 Apr 7;4:e06205. doi: 10.7554/eLife.06205 (PMC4412109; doi:10.7554/eLife.06205)
Supplement: Supplementary file 1. — (A) Yeast Whole Genome Bisulfite Sequencing Data. (B) Yeast MNase Sequencing Stats. (C) Yeast mRNA Sequencing Stats. (D) Yeast ChIP Sequencing Stats. (E) Yeast Whole Genome Bisulfite Sequencing Data for mutant strains. (F) Yeast Whole Genome Bisulfite Sequencing in mouse. (G) ChIP Sequencing Stats in mouse. DOI: http://dx.doi.org/10.7554/eLife.06205.022 [file elife-06205-supp1.docx]

**Supplementary File 1**

**A: Yeast Whole Genome Bisulfite Sequencing Data**

| NAME | STRAIN | GROWTH PHASE | READ LENGTH | # READS | MAPPED READS | MAPPABILITY (%) |
| --- | --- | --- | --- | --- | --- | --- |
| EV strain 1 | W303 | stationary phase | 50 | 5618700 | 4238470 | 75.44 |
| EV_strain 2 | BY4741 | stationary phase | 50 | 5693179 | 4184515 | 73.50 |
| EV strain 3 | W303 | stationary phase | 100 | 18656763 | 13587923 | 72.83 |
| EV_strain 4 | W303 | stationary phase | 100 | 18334345 | 13334549 | 72.73 |
| 3b_exp | W303 | exponential growth | 100 | 30969585 | 20311078 | 65.58 |
| 3b strain 1 | W303 | stationary phase | 100 | 30168485 | 19944982 | 66.11 |
| 3b strain 2 | W303 | stationary phase | 100 | 16815631 | 12460190 | 74.10 |
| 3b strain 3 | W303 | stationary phase | 100 | 18976985 | 14054606 | 74.06 |
| 3b strain 4 | W303 | stationary phase | 100 | 15158680 | 11099360 | 73.22 |
| 3b strain 5 | W303 | stationary phase | 50 | 10538019 | 8543601 | 81.07 |
| 3b strain 6 | W303 | stationary phase | 50 | 11206761 | 9269664 | 82.71 |
| 3b strain 7 | BY4741 | stationary phase | 50 | 11155426 | 8762918 | 78.55 |
| 3b strain 8 | BY4741 | stationary phase | 50 | 8821778 | 7240328 | 82.07 |

**B: Yeast MNase Sequencing Stats**

| NAME | STRAIN | GROWTH PHASE | TYPE | READ LENGTH | # READS | MAPPED READS | | MAPPABILITY (%) | |
| --- | --- | --- | --- | --- | --- | --- | --- | --- | --- |
| EV strain 1 | W303 | stationary | naked-DNA | 50 | 18421958 | | 17828771 | | 96.78 |
| 3b strain 1 | W303 | stationary | naked-DNA | 50 | 19889834 | | 19215569 | | 96.61 |
| EV strain 1 | W303 | stationary | MNase-digested chromatin | 50 | 18746212 | | 18061975 | | 96.35 |
| 3b strain 1 | W303 | stationary | MNase-digested chromatin | 50 | 18536100 | | 17957774 | | 96.88 |

**C: Yeast mRNA Sequencing Stats**

| NAME | STRAIN | GROWTH PHASE | READ LENGTH | # READS | MAPPED READS | MAPPABILITY (%) |
| --- | --- | --- | --- | --- | --- | --- |
| EV strain 3 | W303 | stationary phase | 50 | 17028551 | 14576036 | 85.60 |
| EV_strain 4 | W303 | stationary phase | 50 | 16542825 | 13793873 | 83.38 |
| 3b strain 2 | W303 | stationary phase | 50 | 16739897 | 13835362 | 82.65 |
| 3b strain 3 | W303 | stationary phase | 50 | 16534790 | 13435040 | 81.25 |
| 3b strain 4 | W303 | stationary phase | 50 | 15793246 | 13140669 | 83.20 |

**D: Yeast ChIP Sequencing Stats**

| NAME | SALT (mM) | STRAIN | GROWTH PHASE | READ LENGTH | # READS | MAPPED READS | MAPPABILITY (%) |
| --- | --- | --- | --- | --- | --- | --- | --- |
| polII | 140 | W303 | stationary | 50 | 13558635 | 9428180 | 69.5 |
| DNMT3b | 140 | W303 | stationary | 50 | 11345100 | 7548780 | 66.5 |
| H3K4me1 | 500 | W303 | stationary | 50 | 18396633 | 13745135 | 74.7 |
| H3K4me3 | 500 | W303 | stationary | 50 | 15892023 | 12360992 | 77.8 |
| H3K36me3 | 500 | W303 | stationary | 50 | 16039999 | 12993099 | 81.0 |
| INPUT_1 | 140 | W303 | stationary | 50 | 18552692 | 13700075 | 73.8 |
| INPUT_2 | 500 | W303 | stationary | 50 | 15269649 | 8392024 | 55.0 |

**E: Yeast Whole Genome Bisulfite Sequencing Data for mutant strains**

| NAME | STRAIN | GROWTH PHASE | READ LENGTH | # READS | MAPPED READS | MAPPABILITY (%) |
| --- | --- | --- | --- | --- | --- | --- |
| set1Δ replicate 1 | BY4741 | stationary phase | 50 | 12825798 | 10723394 | 83.6 |
| set1Δ replicate 2 | BY4741 | stationary phase | 50 | 9443269 | 7989638 | 84.6 |
| set2Δ replicate 1 | BY4741 | stationary phase | 50 | 10521217 | 8621585 | 81.9 |
| set2Δ replicate 2 | BY4741 | stationary phase | 50 | 11537314 | 9252601 | 80.2 |
| dot1Δ replicate 1 | W303 | stationary phase | 50 | 11307035 | 9018367 | 79.8 |
| dot1Δ replicate 2 | W303 | stationary phase | 50 | 10711735 | 8624989 | 80.5 |

**F: Whole Genome Bisulfite Sequencing in mouse**

| NAME | | TIME | READ LENGTH | | # READS | | MAPPED READS | | MAPPABILITY (%) | |
| --- | --- | --- | --- | --- | --- | --- | --- | --- | --- | --- |
| E13.5 | E13.5 | | | 50 | | 244054365 | 175922769 | 72.1 | |  |
| E16.5 | E16.5 | | | 100 | | 1080044130 | 750630672 | 69.5 | |  |
| P2.5 | P2.5 | | | 100 | | 621842708 | 416532028 | 67.0 | |  |

**G: ChIP Sequencing Stats in mouse**

| NAME | | TIME | READ LENGTH | | # READS | | MAPPED READS | | | MAPPABILITY (%) |
| --- | --- | --- | --- | --- | --- | --- | --- | --- | --- | --- |
| E16.5 INPUT | E16.5 | | | 50 | | 103447430 | | 37359661 | 36.1 | |
| E16.5 K36me3 IP | E16.5 | | | 50 | | 72711515 | | 28013262 | 38.5 | |
