# Supplementary material for: In vivo targeting of de novo DNA methylation by histone modifications in yeast and mouse
Source: eLife. 2015 Apr 7;4:e06205. doi: 10.7554/eLife.06205 (PMC4412109; doi:10.7554/eLife.06205)
Supplement: Supplementary file 2. — (A) Yeast dinucleotide context methylation. (B) Yeast mutant strains dinucleotide context methylation. (C) Mouse germ cells dinucleotide context methylation. DOI: http://dx.doi.org/10.7554/eLife.06205.023 [file elife-06205-supp2.docx]

**Supplementary File 2**

**A: Yeast dinucleotide context methylation**

| NAME | STRAIN | GROWTH PHASE | 5^me^C CONTEXT (METHYLATION PERCENTAGE) | | | | |
| --- | --- | --- | --- | --- | --- | --- | --- |
|  |  |  | **all** | **CpG** | **CpA** | **CpT** | **CpC** |
| EV strain 1 | W303 | stationary phase | 0.19 | 0.27 | 0.17 | 0.19 | 0.18 |
| EV strain 2 | BY4741 | stationary phase | 0.18 | 0.26 | 0.15 | 0.18 | 0.18 |
| EV strain 3 | W303 | stationary phase | 0.21 | 0.25 | 0.18 | 0.23 | 0.22 |
| EV strain 4 | W303 | stationary phase | 0.23 | 0.26 | 0.20 | 0.25 | 0.24 |
| 3b exp | W303 | exponential growth | 0.81 | 1.76 | 0.77 | 0.55 | 0.54 |
| 3b strain 1 | W303 | stationary phase | 1.51 | 6.00 | 0.91 | 0.56 | 0.54 |
| 3b strain 2 | W303 | stationary phase | 1.21 | 6.08 | 0.43 | 0.28 | 0.25 |
| 3b strain 3 | W303 | stationary phase | 0.94 | 4.52 | 0.35 | 0.26 | 0.24 |
| 3b strain 4 | W303 | stationary phase | 0.86 | 4.06 | 0.32 | 0.26 | 0.24 |
| 3b strain 5 | W303 | stationary phase | 1.47 | 7.73 | 0.55 | 0.29 | 0.24 |
| 3b strain 6 | W303 | stationary phase | 1.44 | 7.65 | 0.58 | 0.30 | 0.25 |
| 3b strain 7 | BY4741 | stationary phase | 0.70 | 3.27 | 0.29 | 0.24 | 0.23 |
| 3b strain 8 | BY4741 | stationary phase | 0.75 | 3.33 | 0.38 | 0.29 | 0.25 |

**B: Yeast mutant strains dinucleotide context methylation**

| NAME | STRAIN | GROWTH PHASE | 5^me^C CONTEXT (METHYLATION PERCENTAGE) | | | | |
| --- | --- | --- | --- | --- | --- | --- | --- |
|  |  |  | **all** | **CpG** | **CpA** | **CpT** | **CpC** |
| set1Δ replicate 1 | BY4741 | stationary phase | 0.34 | 0.84 | 0.27 | 0.25 | 0.23 |
| set1Δ replicate 2 | BY4741 | stationary phase | 0.30 | 0.78 | 0.20 | 0.22 | 0.21 |
| set2Δ replicate 1 | BY4741 | stationary phase | 0.48 | 1.90 | 0.24 | 0.24 | 0.23 |
| set2Δ replicate 2 | BY4741 | stationary phase | 0.52 | 1.97 | 0.31 | 0.27 | 0.25 |
| dot1Δ replicate 1 | W303 | stationary phase | 1.17 | 5.96 | 0.44 | 0.27 | 0.24 |
| dot1Δ replicate 2 | W303 | stationary phase | 1.20 | 6.16 | 0.50 | 0.29 | 0.25 |
| EV strain 1 | W303 | stationary phase | 0.19 | 0.27 | 0.17 | 0.19 | 0.18 |
| EV strain 2 | BY4741 | stationary phase | 0.18 | 0.26 | 0.15 | 0.18 | 0.18 |
| EV strain 3 | W303 | stationary phase | 0.21 | 0.25 | 0.18 | 0.23 | 0.22 |
| EV strain 4 | W303 | stationary phase | 0.23 | 0.26 | 0.20 | 0.25 | 0.24 |

**C: Mouse Germ Cells dinucleotide context methylation**

| NAME | TIME | 5^me^C CONTEXT (METHYLATION PERCENTAGE) | | | | |
| --- | --- | --- | --- | --- | --- | --- |
|  |  | **all** | **CpG** | **CpA** | **CpT** | **CpC** |
| E13.5 | E13.5 | 3.3 | 6.8 | 3.3 | 2.9 | 3.1 |
| E16.5 | E16.5 | 5.1 | 56.2 | 5.8 | 1.8 | 0.7 |
| P2.5 | P2.5 | 7.99 | 77.1 | 9.9 | 2.9 | 0.9 |
