# Supplementary material for: In vivo targeting of de novo DNA methylation by histone modifications in yeast and mouse
Source: eLife. 2015 Apr 7;4:e06205. doi: 10.7554/eLife.06205 (PMC4412109; doi:10.7554/eLife.06205)
Supplement: Supplementary file 5. — Correletion coefficients of DNMT3b occupancy and 5meC levels predictions. DOI: http://dx.doi.org/10.7554/eLife.06205.026 [file elife-06205-supp5.docx]

**Supplementary File 5**

**Correletion coefficients of DNMT3b occupancy and 5^me^C levels predictions**

| PREDICTOR(S) | | | PREDICTED: 5^me^CPG LEVELS | | PREDICTED: DNMT3B OCCUPANCY | |
| --- | --- | --- | --- | --- | --- | --- |
|  |  |  | **CORRELATION** | **ADJ R^2^** | **CORRELATION** | **ADJ R^2^** |
| H3K4me3 | | | 0.675381 | 0.4453 | 0.4309058 | 0.1637 |
| H3K36me3 | | | 0.3837221 | 0.1474 | 0.6271073 | 0.3927 |
| DNMT3b/5^me^C | | | 0.7000648 | 0.4627 | 0.7000648 | 0.4627 |
| nucleosome | | | 0.0715297 | 0.003606 | 0.147471 | 0.02157 |
| RNApolII | | | 0.004768579 | -3.43E-05 | 0.3148327 | 0.1119 |
| H3K4me3 | **H3K36me3** | | 0.7868484 | 0.6114 | 0.7713618 | 0.5749 |
| DNMT3b/5^me^C | **H3K36me3** | | 0.7034986 | 0.4657 | 0.8002787 | 0.6194 |
| H3K4me3 | **H3K36me3** | **nucleosome** | 0.786882 | 0.6114 | 0.7783655 | 0.5832 |
| H3K4me3 | **H3K36me3** | **RNApolII** | 0.7873445 | 0.6134 | 0.7760999 | 0.583 |
| H3K4me3 | **H3K36me3** | **DNMT3b/5^me^C** | 0.8215763 | 0.6635 | 0.8090604 | 0.632 |
| all | | | 0.8247778 | 0.6706 | 0.8221772 | 0.6533 |
