# Supplementary material for: In vivo targeting of de novo DNA methylation by histone modifications in yeast and mouse
Source: eLife. 2015 Apr 7;4:e06205. doi: 10.7554/eLife.06205 (PMC4412109; doi:10.7554/eLife.06205)
Supplement: Supplementary file 6. — (A) Plasmids used in this study. (B) Yeast strains used in this study. (C) Oligonucleotides used in this study. (D) Antibodies used in this study. DOI: http://dx.doi.org/10.7554/eLife.06205.027 [file elife-06205-supp6.docx]

**Supplementary File 6**

**A: Plasmids used in this study**

| PLASMIDS | NAME IN THE PAPER | | | EXPRESSION OF |
| --- | --- | --- | --- | --- |
| pYES2 | | **EV** | **N/A** | |
| pYES2-DNMT3b | | **DNMT3b** | **MmDNMT3b** | |

**B: Yeast strains used in this study**

| PLASMIDS | GENETIC BACKGROUND | | GENOTYPE | |
| --- | --- | --- | --- | --- |
| W303 | | W303 | | MATa, leu2-3,112 trp1-1 can1-100 ura3-1 ade2-1 his3-11,15 |
| BY4741 | | BY4741 | | MATa, his3Δ1, leu2Δ0, met15Δ0, ura3Δ0 |
| *set1*Δ (KLY170) | | BY4741 | | MATa, his3Δ1, leu2Δ0, met15Δ0, ura3Δ0, set1::KAN |
| *set2*Δ (KLY156) | | BY4741 | | MATa, his3Δ1, leu2Δ0, met15Δ0, ura3Δ0, set2::HIS3 |
| *dot1*Δ | | W303 | | MATa, leu2-3,112 trp1-1 can1-100 ura3-1 ade2-1 his3-11,15, dot1::KAN |

**C: Oligonucleotides used in this study**

| NAME | TARGET | SEQUENCE | NOTES |
| --- | --- | --- | --- |
| 3b_Fw | DNMT3b | TAAATATAAA AAGCTT C GGT CCG GCC TCA CGA CAG GAA ACA AT | Used to amplify pCR-BLUNT II-TOPO DNMT3b |
| 3b_Rev | DNMT3b | AATTATTTTA GGATC CGG ACC GTCCCCAGTCCTGGGTAGAAC |  |
| Dot1_UP45 | KanMX | CACCAGTAATTGTGCGCTTTGGTTACATTTTGTTGTACAGTAATGATAACTTCGTATAATGTATGC | Used to amplify kanMX cassette with loxP flanking sequences for PCR-based gene disruption |
| Dot1_DOWN45 | KanMX | CTTAGTTATTCATACTCATCGTTAAAAGCCGTTCAAAGTGCCTCATGATAACTTCGTATAGCATAC |  |
| yTDH1_qFw | TDH1 | TGCTGCTAAGGCTGTCGGTA | qPCR primers |
| yTDH1_qRev | TDH1 | CAACGGCATCTTCGGTGTAA |  |
| mDNMT3b_qFw | DNMT3b | CTGTGGAGTTTCCGGCTACC | qPCR primers |
| mDNMT3b_qRev | DNMT3b | TGCTCTCTGCATCCACCTGT |  |

**D: Antibodies used in this study**

| TARGET | SUPPLIER | | | CATALOG # | LOT # | USED FOR ChIP (μl) |
| --- | --- | --- | --- | --- | --- | --- |
| RNA pol II | | Covance | MMS-126R-200 | | D13HF02305 | 2.5 |
| H3K4me1 | | Abcam | ab8895 | | GR149140-1 | 2.5 |
| H3K4me3 | | Active Motif | 39159 | | 12613005 | 3 |
| H3K36me3 | | Abcam | ab9050 | | GR114293-1 | 4 |
| DNMT3b | | Abcam | ab2851 | | GR101720-2 | 3 |
